# A Principal Components Analysis and Functional Annotation of Differentially Expressed Genes in Brain Regions of Gray Rats Selected for Tame or Aggressive Behavior

**DOI:** 10.3390/ijms25094613

**Published:** 2024-04-23

**Authors:** Irina Chadaeva, Rimma Kozhemyakina, Svetlana Shikhevich, Anton Bogomolov, Ekaterina Kondratyuk, Dmitry Oshchepkov, Yuriy L. Orlov, Arcady L. Markel

**Affiliations:** 1 Institute of Cytology and Genetics SB RAS, Novosibirsk 630090, Russia; ichadaeva@bionet.nsc.ru (I.C.); korimma@bionet.nsc.ru (R.K.); kondratyukey@bionet.nsc.ru (E.K.); markel@bionet.nsc.ru (A.L.M.); 2 Department of Natural Sciences, Novosibirsk State University, Novosibirsk 630090, Russia; 3 Siberian Federal Scientific Centre of Agro-BioTechnologies, Russian Academy of Sciences, Krasnoobsk 630501, Russia; 4 Research Institute of Clinical and Experimental Lymphology—Branch of Institute of Cytology and Genetics, Novosibirsk 630117, Russia; 5 Institute of Biodesign and Complex Systems Modeling, Sechenov First Moscow State Medical University (Sechenov University), Moscow 119991, Russia; 6 Agrarian and Technological Institute, Peoples’ Friendship University of Russia, Moscow 117198, Russia

**Keywords:** animal domestication, artificial selection, behavioral genetics, brain, differentially expressed gene, aggressive and tame rats, RNA-seq, principal component analysis, functional annotation of genes, gene network

## Abstract

The process of domestication, despite its short duration as it compared with the time scale of the natural evolutionary process, has caused rapid and substantial changes in the phenotype of domestic animal species. Nonetheless, the genetic mechanisms underlying these changes remain poorly understood. The present study deals with an analysis of the transcriptomes from four brain regions of gray rats (*Rattus norvegicus*), serving as an experimental model object of domestication. We compared gene expression profiles in the hypothalamus, hippocampus, periaqueductal gray matter, and the midbrain tegmental region between tame domesticated and aggressive gray rats and revealed subdivisions of differentially expressed genes by principal components analysis that explain the main part of differentially gene expression variance. Functional analysis (in the DAVID (Database for Annotation, Visualization and Integrated Discovery) Bioinformatics Resources database) of the differentially expressed genes allowed us to identify and describe the key biological processes that can participate in the formation of the different behavioral patterns seen in the two groups of gray rats. Using the STRING- DB (search tool for recurring instances of neighboring genes) web service, we built a gene association network. The genes engaged in broad network interactions have been identified. Our study offers data on the genes whose expression levels change in response to artificial selection for behavior during animal domestication.

## 1. Introduction

The selection of animals showing tame or aggressive behavior toward humans has become the basis of research on the effects and mechanisms of animal domestication. This work was started by D.K. Belyaev back in 1958 on farm foxes [1]; later, it was continued on minks and gray rats and is unique in duration, scale, and complexity. It has been shown that alterations of behavior from the “wild” to “tame” type follow general patterns and are on the spectrum of manifestations of so-called domestication syndrome [2,3,4].

At the genotype level, the adaptation of animals to captive conditions is a relatively slow and species-specific process; it can be detected only after several dozen generations. Therefore, experimental modeling of the domestication process requires a lot of time. Currently, there are only a few examples of the experimental-domestication research [5,6] whose results—with certain limitations—are commensurate with the findings of the large-scale comprehensive experiment of D.K. Belyaev and his followers at the Institute of Cytology and Genetics of Siberian Branch of the Russian Academy of Sciences (SB RAS) in Novosibirsk, Russia [7,8]. Nevertheless, the data obtained so far are fragmentary and do not show the overall picture, whereas the identification of the genes associated with tame or aggressive behavior toward humans (and of combinations of their mutual effects on the manifestation of behavior) is truly an extraordinary task.

Studies on gene expression in the brains of wild and domestic animals are sparse and are mainly focused on the cerebral cortex [7,9], whereas other brain regions (involved in emotional reactions, a stress response, or neurogenesis) have been investigated to a lesser extent and are likely to be relevant to alterations of behavior under artificial selection pressure. On the other hand, animal behavior is controlled by a coordinated work of neurons from different brain regions (see a very detailed review [10]), and therefore such data should be analyzed comprehensively.

The lines of the tame and aggressive gray rats were developed at the Institute of Cytology and Genetics of the SB RAS in Novosibirsk having an aim to study the dramatic behavioral changes in animals subjected to domestication. After all, the domestication of animals is characterized by a gradual decrease in aggressiveness toward humans. Gray rats, which have undergone long-term selection for behavioral response toward humans, serve as a unique model for analyzing the complex genetic changes underlying the behavioral transformations during animal domestication. In tame and aggressive rats, it is possible to correctly and adequately assess the behavioral manifestations of aggressive or non-aggressive response to humans by the glove test (a hand in a glove placed into the cage [11]).

From the very first works on the artificial selection for behavior of wild-caught gray rats to the present, a large amount of data in our and in collaborators laboratories has been obtained on physiology [11,12,13,14], behavior [15,16,17], and genetic differences [18,19,20]. Previously, we have investigated gene expression profiles in four brain regions of tame and aggressive gray rats: the hypothalamus [21], the hippocampus [22], the periaqueductal gray matter (PAG) [23], and the tegmental region of the midbrain (MTg) [24]. Based on these profiles, we found 46, 42, 39, and 42 differentially expressed genes (DEGs), respectively. By combining these four sets of DEGs from the brain regions of tame and aggressive rats and by removing duplicate gene names, we obtained a total list of 112 DEGs in the present work. Our complex analysis offers data on genes that change expression in response to artificial selection for behavior, and these findings can be used in animal breeding in agriculture and veterinary medicine.

## 2. Results

### 2.1. Examination of the List of DEGs from Brain Samples of Tame and Aggressive Rats by Principal Component Analysis (PCA)

**Figure 1:**
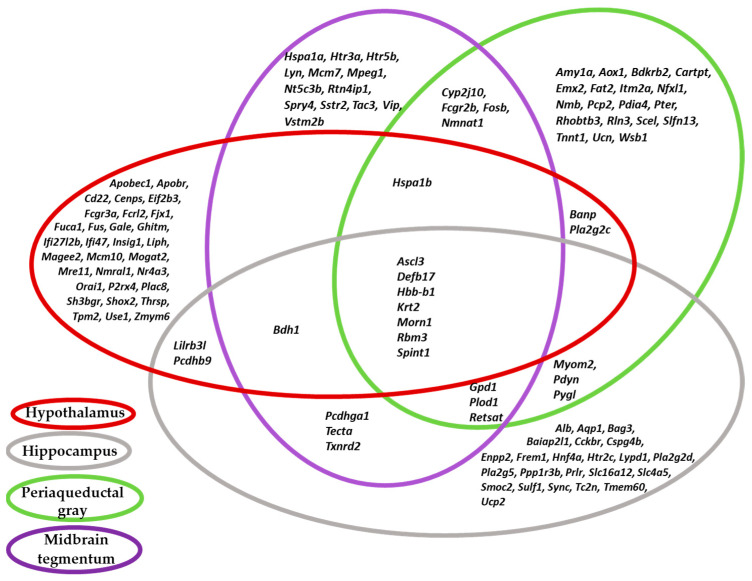
The Venn diagram depicting overlaps of differentially expressed genes (DEGs) sets among the four analyzed brain regions of tame and aggressive rats.

On the basis of previously published transcriptomes of the hypothalamus, hippocampus, PAG, and MTg of gray rats selected for their reaction to humans, we compiled a total list of DEGs, which consisted of 112 genes. The Venn diagram below shows overlaps of DEG sets among the four brain regions (Figure 1).

The DEG sets from the different brain regions partially overlapped with one another, and the overlaps varied from 9 genes (between the hypothalamus and MTg: genes *Ascl3*, *Bdh1*, *Defb17*, *Hbb-b1*, *Hspa1b*, *Krt2*, *Morn1*, *Rbm3*, and *Spint1*) to 15 genes (between the midbrain regions [the PAG and MTg]: genes *Ascl3*, *Bdh1*, *Cyp2j10*, *Defb17*, *Fcgr2b*, *Fosb*, *Gpd1*, *Hbb-b1*, *Hspa1b*, *Krt2*, *Morn1*, *Nmnat1*, *Plod1*, *Rbm3*, and *Spint1*). It is important to note that changes in the expression of the same DEGs in several brain regions are unidirectional: that is, they rather depend on the direction of selection than on the brain localization. For example, in the hypothalamus, the *Ascl3* gene is more strongly expressed in tame rats, and the same is true for the hippocampus, PAG, and MTg, whereas the expression of the *Banp* gene is higher in the hypothalamus and PAG of the aggressive animals. These coincidences in the differential expression of genes in different brain regions of the two rat lines indirectly indicate connections between these genes in how they implement a unidirectional regulation of behavior at the genetic level.

**Figure 2:**
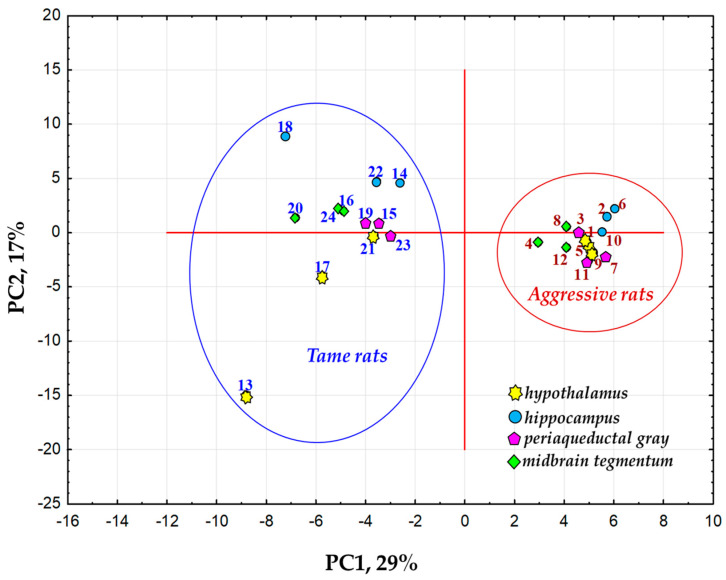
The distribution of brain region samples from tame and aggressive rats by levels of DEGs’ expression (112 genes in total) in these samples with the first (PC1) and second (PC2) principal components as coordinates. The right-hand cluster of the graph combines brain samples from three aggressive rats (samples numbered 1–12), and the left-hand cluster contains brain samples from three tame rats (samples numbered 13–24).

The data on differential gene expression in the four brain regions of tame and aggressive rats were then subjected to principal component analysis (PCA). It revealed a first principal component (here and further: PC1), which explained the largest proportion of variance of gene expression in the brain regions between the two lines (29%) and a second principal component (here and further: PC2; 17% of the variance; Figure 2).

**Figure 3:**
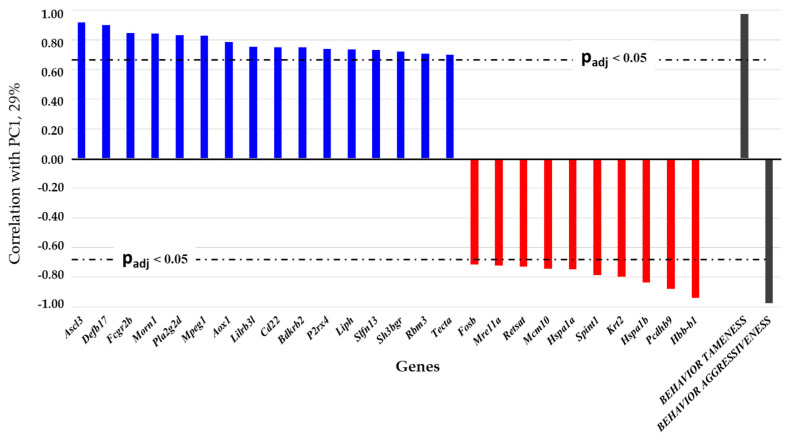
A statistically significant linear correlation between expression levels (expressed in absolute values of FPKM) of DEGs and the first principal component, PC1. Genes whose expression levels are higher in tame rats are highlighted in blue; the genes whose expression is higher in aggressive rats are highlighted in red. As an auxiliary tool, indicator functions were employed: “tame behavior”, which equals 1.0 for tame rats and 0 for aggressive ones, and “aggressive behavior”, which equals 1.0 for the aggressive animals and 0 for the tame ones. The dash-and-dot lines show boundaries of the 95% confidence interval.

As for the PC1, the rats were subdivided into two groups on the basis of the 26 genes (out of 112 DEGs) that showed a significant correlation (correlation coefficients of 0.70 to 0.92 and of −0.71 to −0.94 with Bonferroni’s correction, p_adj_ < 0.001) with this principal component, which reflected 29% of the total variance. Overall, 16 out of these 26 genes are upregulated in tame rats, and 10 others are upregulated in the aggressive animals (Figure 3).

The PC1 reflects gene expression changes that are caused by artificial selection based on the behavioral response of rats to humans (glove test [11]). Accordingly, this component’s positive coefficient of correlation with genes *Ascl3*, *Defb17*, *Fcgr2b*, *Morn1*, *Pla2g2d*, *Mpeg1*, *Aox1*, *Lilrb3l*, *Cd22*, *Bdkrb2*, *P2rx4*, *Liph*, *Slfn13*, *Sh3bgr*, *Rbm3*, and *Tecta* points to a connection with the tame behavioral phenotype, and the negative correlation with genes *Fosb*, *Mre11a*, *Retsat*, *Mcm10*, *Hspa1a*, *Spint1*, *Krt2*, *Hspa1b*, *Pcdhb9*, and *Hbb-b1* indicates an association with the rat phenotype of the aggressive response (see Figure 3). The PC1 can be designated as a “behavioral determination of the gene expression in the brain of tame and aggressive rats”. Thus, it is changes in the expression of these 26 genes that contribute to the behavioral phenotype of the two rat lines.

**Figure 4:**
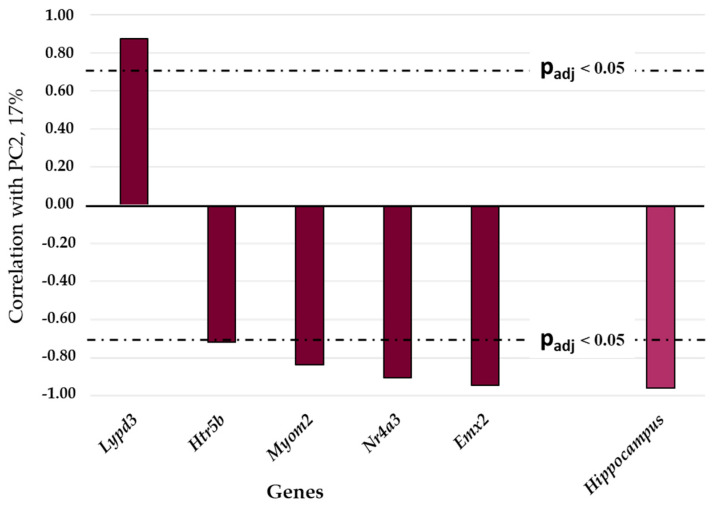
The statistically significant correlation between expression levels (expressed in absolute values of FPKM) of DEGs and the second principal component, PC2. As an auxiliary tool, indicator function “Hippocampus” was employed. The dash-and-dot lines denote boundaries of the 95% confidence interval.

The contribution of the PC2 accounted for only 17% of the total variance of gene expression under our experimental conditions. This principal component correlates with the expression levels of five genes in the hippocampus: *Lypd1* (r = 0.87 with the Bonferroni correction, p_adj_ < 0.001), *Htr5b* (r = −0.72), *Myom2* (r = −0.84), *Nr4a3* (r = −0.91), and *Emx2* (r = −0.95; Figure 4).

The PC2 reflects hippocampus-specific changes of gene expression in tame and aggressive rats: four genes out of the five listed above show significant upregulation in the hippocampus of both tame and aggressive rats. These genes negatively correlate with PC2. Accordingly, a positive correlation coefficient for the *Lypd1* gene corresponds to statistically significant underexpression in the hippocampus of gray rats of both lines. For the other three brain regions under study (the hypothalamus, PAG, and MTg) of rats of both lines, no correlations with PC2 were found. It can be concluded that the expression of some DEGs of tame and aggressive rats can substantially depend on a brain region in which they are active: in this case, the hippocampus. Therefore, the PC2 can be designated as “regional determination of gene expression in the brain of tame and aggressive rats”.

For the other principal components, it was found that their values are within the 95% confidence intervals, and they were not included in further analyses.

Thus, the genetic effects of artificial selection of gray rats for behavior are associated with differences in the expression levels of 112 genes in the hypothalamus, hippocampus, PAG and MTg, of which the increased expression of 16 genes is associated with the tame behavior phenotype and the increased expression of 10 genes is with aggressive behavior. In addition, changes in the expression of five genes (*Lypd1*, *Htr5b*, *Myom2*, *Nr4a3* and *Emx2*) are due to regional features of gene functioning in the hippocampus of animals of both lines.

### 2.2. Functional Annotation of DEGs in the Brain Regions of Tame and Aggressive Rats

This annotation was carried out using web service DAVID Bioinformatics Resources (https://david.ncifcrf.gov/ (accessed on 10 March 2024)) and included an analysis of biological processes. Then, the enrichment of the DEG list with categories of KEGG PATHWAY and Gene Ontology (GO) was calculated for the transcriptome data of tame and aggressive rats as background and for the gray-rat genome as background (installed in DAVID by default); identical results were obtained. This analysis allowed us to determine which biological processes and ontology terms could lead to the changes of the behavioral response toward humans in rats of the two lines (tame and aggressive; Table 1).

Table 1 shows that the top gene ontology terms were related to signal transduction. The most statistically significantly enriched term was “Neuroactive ligand–receptor interaction” (*p* < 0.0029), which was associated with 12 genes. Six of them had higher levels of expression in the brain regions of tame rats (*Bdkrb2*, *Cckbr*, *Htr2c*, *Prlr*, *P2rx4*, and *Ucn*), and the other six were expressed more strongly in the aggressive animals (*Nmb*, *Pdyn*, *Rln3*, *Sstr2*, *Tac3*, and *Vip*).

The second most significant GO term was “Cleavage on pair of basic residues” (*p* < 0.008), which is related to eight DEGs (*Alb*, *Cartpt*, *Enpp2*, *Pdyn*, *Rln3*, *Tac3*, *Ucn*, and *Vip*). The third most significant GO term was found to be “Arachidonic acid secretion” (*p* < 0.0083), which was associated with the smallest number genes: they encode a group of phospholipase enzymes, a neuromedin B, and bradykinin receptor: five genes from the total list of DEGs (*Bdkrb2*, *Nmb*, *Pla2g2c*, *Pla2g2d*, and *Pla2g5*).

The largest number of DEGs proved to be related to GO terms “Signal” (*p* < 0.021, 38 genes, including important ones for behavior analysis: *Alb*, *Cartpt*, *Htr2c*, *Htr3a*, and others), and “Disulfide bond” (*p* < 0.044, 27 genes). In the Signal gene set, expression levels of 25 out of 38 DEGs were higher in tame rats compared to aggressive ones (Table 1). DEGs of the Signal gene set included genes of various functions of encoded proteins, such as widely known and well-studied receptor genes (serotonin receptor genes *Htr2c* and *Htr3a* and prolactin receptor gene *Prlr*), enzyme genes of various metabolic pathways (*Liph*, *Pla2g2c*, *Pla2g2d*, *Pla2g5*, and *Sulf1*), and others. Thus, we can conclude that in gray rats, the selection for the reaction to humans has led to changes in the expression of genes taking part in the signaling activity of neurons.

Overall, 8 out of 27 DEGs related to GO term “Disulfide bond” from the chemical bond category have a higher level of expression in aggressive rats, and accordingly, 19 genes are expressed more strongly in tame ones. The GO term “Extracellular region” was poorer in the number of associated DEGs (*p* < 0.021); this DEG set contained 16 genes: *Alb*, *Defb17*, *Enpp2*, *Hspa1a*, *Hspa1b*, *Liph*, *Nmb*, *Pla2g2c*, *Pla2g2d*, *Pla2g5*, *Pdyn*, *Rln3*, *Tac3*, *Tecta*, *Ucn*, and *Vip*. The proteins of these genes are involved in various extracellular processes by mediating necessary interactions between cells. The expression levels of eight genes from this set are higher in tame rats (*Alb*, *Defb17*, *Enpp2*, *Liph*, *Pla2g2d*, *Pla2g5*, *Tecta*, and *Ucn*), and the other eight are expressed more strongly in aggressive rats (*Hspa1a*, *Hspa1b*, *Nmb*, *Pla2g2c*, *Pdyn*, *Rln3*, *Tac3*, and *Vip*). Based on this result, we can theorize that in the system of intercellular interactions, rats of the two behaviorally contrasting lines differ not in the number of upregulated genes but rather in the effects of these genes’ products.

The GO term “Lipid metabolism” (*p* < 0.01) was found to be related to 12 DEGs and is interesting in that almost all these genes have a higher level of expression in tame rats compared to aggressive ones. Overall, 11 of the 12 genes in this DEG set encode various enzymes participating in lipid metabolism. In contrast, the *Thrsp* gene (thyroid hormone responsive), the remaining 1 out of 12 in this category, does not code for an enzyme. Its protein THRSP is presumably involved in lipid metabolism and biosynthesis, and its expression is under the control of thyroid hormones [25].

### 2.3. Gene Association Networks Describing Interactions of DEGs in the Brain Regions of Tame and Aggressive Rats

**Figure 5:**
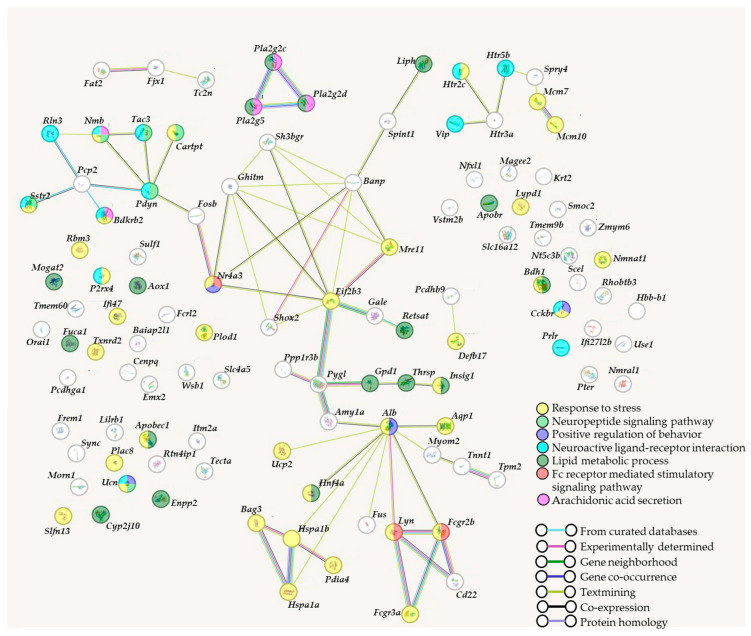
The gene association network built by means of web service STRING-DB on the basis of protein–protein interactions corresponding to some DEGs of tame and aggressive rats. Genes that are related to one of seven biological processes are highlighted in a color(s), and interactions between genes are indicated by lines.

To identify possible relations between some DEGs (found in brain regions of gray rats with tame or aggressive behavior), a gene association network was built using the STRING-DB tool (https://string-db.org) (accessed on 18 January 2024) (Figure 5).

Figure 5 presents several groups of DEGs that are linked by functional connections (e.g., co-expression, protein homology, or textmining). For instance, there is a group where serotonin receptor genes (*Htr2c*, *Htr5b*, and *Htr3a*; Figure 5) are connected with genes *Vip* (vasoactive intestinal peptide) and *Spry4* (Sprouty RTK-signaling antagonist 4) and two genes *Mcm7* and *Mcm10* whose proteins initiate DNA replication. In this group, genes are united by joint expression and close localizations in the genome. Interactions between genes *Mcm7* and *Mcm10* are confirmed experimentally and indicate homologous encoded proteins.

We have varied parameters of interactions between gene products for the network reconstruction counting only protein interaction or gene co-expression and textmining (Figure 5). Still the network is rather sparse, indicating the diverse effects of the genes under analysis. The gene network contains one large cluster, which includes such relevant genes as *Pcp2* (Purkinje cell protein 2), *Nmb* (neuromedin B), *Fosb* (a Fos family transcription factor), *Nr4a3* (a nuclear receptor and transcription factor), *Shox2* (short-stature homeobox 2, a transcription factor), *Hspa1a* and *Hspa1b* (heat shock protein genes) and others: a total of 77 DEGs out of 112. In this total list, 33 DEGs were found to not participate in any interactions. As a result, a functional analysis of the list of 112 DEGs in the hypothalamus, hippocampus, PAG, and MTg showed the presence of gene network structures that determine differences in the behavior of tame and aggressive rats. In the brain regions studied, the significant interactions have been identified for the transcription factor (*Fosb*) and serine peptidase inhibitor (*Spint1*) genes; the stress response genes (*Bdkrb2*, *Hspa1b* and *Hspa1a*); the genes involved in the regulation of neurochemical (*Slfn13*, *P2rx4*, *Rbm3* and *Sh3bgr*) and immune processes (*Defb17*, *Cd22* and *Fcgr2b*); the genes of lipid metabolism (*Aox1*, *Retsat*, *Pla2g2d* and *Liph*), and the genes of DNA reparation (*Mre11a*) and replication (*Mcm10*).

In the presented gene association network, several clusters correspond to sets of genes grouped by biological processes in the DAVID database (Table 1). For instance, in both databases (DAVID and STRING), the term “Arachidonic acid secretion” is related to genes *Bdkrb2*, *Nmb*, *Pla2g2c*, *Pla2g2d*, and *Pla2g5*. The term “Lipid metabolic process” is associated with genes *Aox1*, *Bdh1*, *Fuca1*, *Enpp2*, *Insig1*, *Liph*, *Mogat2*, *Pla2g2c*, *Pla2g2d*, *Pla2g5*, *Retsat*, and *Thrsp* according to DAVID, whereas according to STRING, this term is additionally related to genes *Apobec1*, *Apobr*, *Cyp2j10*, *Gpd1*, and *Hnf4a*. During the analysis in the STRING software (version 12.0), additional clusters were identified such as “Positive regulation of behavior”, “Fc receptor-mediated stimulatory signaling pathway”, and “Response to stress”.

Thus, the DAVID and STRING functional enrichment analysis results complement each other and identify overrepresented terms that drive behavioral responses. All this together determines a multifactor system controlling the two different types of behavior, tame or aggressive toward human, for which selection is carried out.

## 3. Discussion

In this work, we analyzed previously published transcriptomes observed in the brain regions of tame and aggressive rats, in which 46 DEGs were identified in the hypothalamus [21], 42 in the hippocampus [22], 39 in the PAG [23], and 42 in the MTg [24]. Both functional annotations using standard approaches and principal component analysis were performed to identify the number and nature of significant factors contributing to differences in the gene expression. Although a number of methods for quantifying the contribution of known sources of variation in complex gene expression studies are available (e.g., see [26]), our aim was to find the hypothetical variables (components) accounting for as much as possible of the existing variance in the gene expression data, i.e., to perform principal component analysis. Further possibility to test the components significance via a bootstrapping procedure [27] allows us to assert that we have taken into account all the significant components.

### 3.1. Relations of DEGs in the Brain Regions of Gray Rats with Behavior According to the PC1 and PC2

We showed that 26 DEGs of tame and aggressive rats are associated with behavioral differences: this is the gene expression variation factor represented by PC1, which was designated as a “behavioral determination of gene expression in the brain of tame and aggressive rats”. Five other DEGs are associated with the factor “regional determination gene expression in the brain of tame and aggressive rats” (PC2), which should be considered as a factor of specific genetic regulation of hippocampal function. It should be pointed out that peculiarities of gene expression in different brain regions that perform similar functions (for example, in our research, the brain regions were specifically involved in control of aggressive behavior) are not obvious. This is because within our study, gene expression products are participate in similar biological processes [28,29]. At the same time, the magnitude of influence of the PC2 on the overall variation of the expression of all the examined genes is only marginally statistically significant; therefore, we cannot claim with certainty that the level of expression of the investigated genes is specific just to the brain region.

The DEGs analyzed in the summed four brain regions of tame and aggressive gray rats from the 90th generation of selection are connected to the implementation of diverse biological processes, thereby indicating a large set of genes underlying the differences in the studied behavioral phenotypes between the groups of animals being compared. The statistically significant difference in expression levels of all the genes that we identified during the comparison of expression data between tame and aggressive rats (i.e., the total list of DEGs) indicates considerable biochemical and metabolic differences in the functioning of the central nervous system, directly or indirectly resulting in a change in the behavioral repertoire of the groups of animals being compared. Out of the 112 DEGs, the expression levels of seven (*Ascl3*, *Defb17*, *Hbb-b1*, *Krt2*, *Morn1*, *Rbm3*, and *Spint1*) differed significantly between the two groups of animals in all four examined brain regions (Figure 1). This finding can be compared with the data obtained by Sato and coworkers (2020) in domestic and wild rabbits, where 27 out of 612 DEGs were found whose changes in expression turned out to be common among selected parts of the brain (amygdala, hypothalamus, hippocampus, and parietal/temporal cortex) [30]. This observation seems to be a convincing explanation of the differences in behavioral reactions to humans and in the phenotype as a whole among the studied animals if we take into account that the history of domestication of rabbits is relatively recent (on an evolutionary scale). Regarding tame and aggressive rats, it is obvious that artificial selection during an even shorter period (50 years) has not yet caused such profound genetic differences between these lines. Nevertheless, this period was enough to engender significant differences both in behavior and in brain gene expression.

Expression changes are unidirectional among all the four brain regions for each of these seven genes: expression levels of *Ascl3*, *Defb17*, *Morn1*, and *Rbm3* are significantly higher, whereas the expression levels of genes *Hbb-b1*, *Krt2*, and *Spint1* are significantly lower in tame rats compared to aggressive ones [21,22,23,24]. These DEGs ended up in two groups formed by subdivision according to the PC1 (see Figure 3). Among the above genes, *Ascl3* from the achaete–scute complex-like family (*Ascl*) is highly relevant. This family includes transcription factors (TFs) partaking in the development of the nervous system [31,32,33]. Earlier, by bioinformatic methods, we have examined a relation between the *Ascl3* overexpression in tame rats and the expression changes of other DEGs and revealed that DNA motifs corresponding to potential binding sites of this TF (ASCL3) constitute a noticeable proportion of all motifs significantly enriched in the set of genes underexpressed in the tame rats [24]. The *Ascl3* gene was not found to be associated with any DAVID and STRING ontology categories, which was probably due to the current lack of detailed research on this gene. Nonetheless, our hypothesis about an important role of the TF ASCL3 as a regulator of the genetic basis of the behavioral phenotype in the gray rats being subjected to selection is supported by a study by Benítez-Burraco and colleagues [34]. In their meta-analysis of published lists of DEGs from different species of domestic animals, those authors came to the following conclusion: TF genes influence large sets of genes whose products participate in the development of the brain and of craniofacial morphology. Biological processes regulated by such TFs are mostly involved in determination of the traits that change in mammals under the influence of domestication [34].

The other six genes out of the seven that are differentially expressed between rats of the two lines in all four brain regions also correlate with the PC1 (see Figure 3). Nevertheless, only the *Defb17* gene (β-defensin 17) proved to be associated with several GO terms during functional annotation in the DAVID database (the term “Extracellular region” combining genes by localization of their expression products, the term “Signal” combining genes by relevant function, and the term “Disulfide bond”, which unites genes by protein structure), which is consistent with our analysis in the STRING software, where *Defb17* was found to be among the genes related to the response to stress.

### 3.2. Relations of DEGs in the Brain Regions of Tame and Aggressive Rats with GO and KEGG Terms

Products of DEGs associated with the most statistically significantly enriched term “Neuroactive ligand–receptor interaction” affect neuron function by binding to intracellular and membrane receptors and are related to such processes as “ion transmembrane transport”, “positive regulation of synapse assembly”, “chloride transmembrane transport”, “sodium ion transport”, and “potassium ion transport” [35]. The downregulation of genes of this “Neuroactive ligand–receptor interaction” group impairs memory function [36]. In particular, the *Bdkrb2* gene (a member of the set of genes associated with the PC1 and with the behavioral phenotype, see Figure 3) encodes bradykinin receptor (B2R), which is a key player in neuroprotective activity and synaptic plasticity mediated by neurotrophic nerve growth factor in brain cells; *Cckbr* codes for the cholecystokinin B receptor, which is involved in the neurotransmission performed by GABAergic interneurons; the *Htr2c* gene encodes serotonin receptor 2C, which triggers various signaling pathways; the *Nmb* gene, coding for neuromedin B, causes a release of adrenocorticotropic hormone (ACTH) and corticosterone; the *Pdyn* gene encodes a peptide called prodynorphin, which activates opioid receptor binding and partakes in chemical synaptic transmission and neuropeptide signaling; the *Prlr* gene encodes the prolactin receptor; *P2rx4* codes for purinergic receptor P2RX4, which modulates neurotransmission and synaptic strengthening; the product of *Rln3* (relaxin 3) is localized to the synaptic cleft and acts as a neurotransmitter; the *Sstr2* gene codes for somatostatin receptor 2, which plays neuroendocrine and neurotransmitter roles; the *Tac3* gene encodes tachykinin precursor 3, representing one of the largest classes of neuropeptides; the *Ucn* gene codes for neuropeptide urocortin belonging to the corticotropin-releasing factor protein family; and the *Vip* gene produces vasoactive intestinal peptide, which is a neuropeptide hormone.

The GO term “Lipid metabolism” was associated with 12 genes whose proteins are important participants in lipid metabolism reactions. Via variations in the composition of the plasma membrane, lipid metabolism affects the velocity of nerve impulse transmission: for example, with an increase in the cholesterol content of the membrane, impulse transmission slows down [37]. It is also noteworthy that a component of lipid metabolism is the metabolism of steroid hormones, e.g., testosterone and corticosterone, the levels of which differ between male tame and aggressive rats [38].

The GO term “Signal” combined 38 DEGs out of the 112 found between tame and aggressive rats (Table 1); this finding indicates a critical role of signal transduction in the formation of the behavioral phenotype of the animals in question.

The products of 16 genes related to the term “Extracellular region”—*Alb* (albumin), *Defb17* (β-defensin 17), *Enpp2* (autotaxin), *Hspa1a* and *Hspa1b* (heat shock proteins), *Liph* (lipase H), *Nmb* (neuromedin B), *Pdyn* (prodynorphin), *Pla2g2c*, *Pla2g2d*, and *Pla2g5* (phospholipases), *Rln3* (relaxin 3), *Tac3* (tachykinin precursor 3), *Tecta* (α-tectorin), *Ucn* (urocortin), and *Vip* (vasoactive peptide)—participate in biochemical processes of the extracellular region (ECR). Molecules of the ECR are known to be diffusely distributed throughout the brain in and between synaptic clefts and to facilitate signal transmission between neurons and glial cells by incorporating their secreted signaling molecules into the extracellular space where they interact with other molecules of the ECR and/or cell surface receptors [39]. Of the 16 aforementioned genes, five (*Defb17*, *Hspa1b*, *Liph*, *Pla2g2d*, and *Tecta*) correlated with the behavioral phenotype of gray rats according to our principal component analysis (see Figure 3), thereby confirming a major role of the ECR in the behavior of animals.

As for the term “Arachidonic acid secretion”, in mammals, arachidonic acid is present as a lipid in the brain, liver, and milk fat. Arachidonic acid controls the fluidity of the plasma membrane, affects the function of specific membrane proteins involved in cell signaling, and plays a fundamental role in the maintenance of the integrity of cells and organelles and in vascular permeability. These properties may explain the crucial role of arachidonic acid in the neuron function, synaptic plasticity of the brain, and long-term potentiation in the hippocampus [40]. The three genes of phospholipases A2 associated with this GO term encode enzymes helping to generate arachidonic acid in the cell. The *Pla2g2c* gene is more strongly expressed in aggressive rats in the hypothalamus and PAG, whereas the expression of genes *Pla2g2d* and *Pla2g5* is higher in the hippocampus of tame rats. Nevertheless, only the *Pla2g2d* gene is associated with the PC1, which explains changes in gene expression depending on the behavioral phenotype (see Figure 3). Consequently, our data revealed a connection between the expression of genes participating in the synthesis of arachidonic acid and the behavior of tame and aggressive rats toward humans.

After the construction of the gene association network in the STRING software, it was found (Figure 5) that slightly less than half of the genes ended up in one cluster, in which nodes are represented by *Alb* (albumin), *Amy1a* (amylase alpha 1A), *Banp* (Btg3-associated nuclear protein), *Eif2b3* (eukaryotic translation initiation factor 2B subunit gamma), *Fcgr2b* (Fc [fragment crystallizable] gamma receptor 2B), *Ghitm* (growth hormone-inducible transmembrane protein), *Hspa1b* (heat shock protein family A [Hsp70] member 1B), *Lyn* (LYN proto-oncogene, Src family tyrosine kinase), *Mre11* (MRE11 homolog A, double-strand break repair nuclease), *Nr4a3* (nuclear receptor subfamily 4, group A, member 3), *Pdyn* (prodynorphin), *Pcp2* (Purkinje cell protein 2), *Pygl* (glycogen phosphorylase L), *Sh3bgr* (SH3 domain-binding glutamate-rich protein), and *Shox2* (short-stature homeobox 2). Three genes of phospholipases A2 and serotonin receptor genes formed distinct groups (Figure 5). Genes *Htr3a* and *Htr2c* are more strongly expressed in tame rats in the MTg and hippocampus, respectively, whereas the expression of gene *Htr5b* is higher in the MTg of aggressive rats. According to Ensembl data (http://www.ensembl.org/Rattus_norvegicus/Info/Index, accessed on 18 January 2024), there are 14 genes in the serotonin receptor family in gray rats. In our study, the differential expression here was documented only for 3 of these 14 genes; for the remaining genes of this family, expression differences were not found even at marginal significance. Neurotransmitter serotonin also affects aggression [41,42], but data on the correlation of serotonin levels with aggression are contradictory [43]. A long-term release of serotonin is positively associated both with normal aggressive behavior (territorial conflicts or the establishment of a social hierarchy [41,44]) and with pathophysiological manifestations of this behavior that accompany mental disorders [45]. It has been reported that the amount of *Htr1a* mRNA in the midbrain of adult tame rats is elevated too, although no such differences have been noted between rat pups of the two lines [14]. In 3-month-old tame rats of the 85th generation of selection, the mRNA level of another gene (*Htr7*) is increased in the midbrain and hypothalamus as compared to their aggressive relatives [20]. Thus, it can be concluded that interactions of serotonin receptors are an important regulator of genetically determined aggression: the expression of these genes determines tame and aggressive behavior in gray rats in a complicated and ambiguous manner. Specific mechanisms of this regulation can be identified by detailed research.

Our analysis of the obtained gene association networks points to the existence of an extensive system of genetic regulation of brain functions that has formed as a consequence of the selection of gray rats for behavior. The differences in expression of genes of this network between tame and aggressive rats confirm the importance of this gene network (as a complex genetic regulator) for determining complex types of contrasting behavior in the lines of rats being compared. Genes featuring a large number of interactions can serve as hub regulators of a given gene network. We should highlight the significance of expression changes of the genes that showed significant correlations with the PC1, which we designated as the “behavioral determination of the gene expression in the brain of tame and aggressive rats”. Among these genes are a TF gene (*Fosb*), genes responsible for the stress response (*Bdkrb2*, *Fosb*, *Hbb-b1*, *Hspa1a*, *Hspa1b*, and *Pcdhb9*), genes encoding direct regulators of neurochemical processes (*Morn1*, *P2rx4*, *Rbm3*, *Slfn13*, *Sh3bgr*, and *Tecta*), genes whose products are regulators of immune processes (*Defb17*, *Fcgr2b*, *Mpeg1*, *Lilrb3l*, and *Cd22*) and of DNA metabolism (*Liph*, *Pla2g2d*, and *Retsat*), of DNA repair (*Mre11a*), and of DNA replication (*Mcm7* and *Mcm10*).

The relation—with the tame/aggressive behavioral phenotype—of the 26 genes, as evidenced by PC1, is especially important to examine in the context of functional annotation in GO terms. In this case, grouping into categories enables us to determine which biological processes could cause (or influence) changes in the behavior of animals. After functional annotation of the 26 DEGs correlating with the PC1 (Figure 3), it was evident that not all of them matched GO or KEGG terms. For example, according to the DAVID data, only two genes—*Bdkrb2* and *P2rx4*—are related to the KEGG term “Neuroactive ligand–receptor interaction”, and 10 out of the 26 DEGs belong to the term “Signal”. Genes *Mcm10*, *Mre11a*, *Pcdhb9*, *Slfn13*, and *Sh3bgr* did not match any of the DAVID categories, whereas genes *Aox1*, *Lilrb3l*, *Mpeg1*, *P2rx4*, *Slfn13*, and *Tecta* are not connected via interactions with the examined DEGs according to the STRING database. Genes *Ascl3*, *Rbm3*, *Krt2*, *Hbb-b1*, and *Morn1*—all those (except for *Defb17* and *Spint1*) whose expression differs between rats of the two lines simultaneously in all four brain regions—are not associated with any GO or KEGG categories.

Therefore, among the DEGs identified by us, half are statistically significantly associated with GO or KEGG terms according to publicly available Web database services. That the other DEGs are not engaged in interactions suggests that they are not partners of each other according to any criteria in the gene association network in question. We were able to identify specific genes that differ in expression between rats showing opposite behaviors toward humans. These results can serve as an adequate basis for further research into genetic factors of domestication.

## 4. Materials and Methods

### 4.1. Materials

The results of analysis presented in this work are based on previously published data [21,22,23,24] on genome-wide gene profiling in the brain regions of tame and aggressive gray rats. Lines of the tame and aggressive rats were developed at the Institute of Cytology and Genetics by genetic selection for reduced and increased aggressivity toward humans. The rat’s behavior was tested with the ordinary glove test [13]. If it is possible to pick up aggressive rats in hands protected by thick leather gloves, into which the animals cling with a “dead” grip, then tame rats not only do not avoid, but, on the contrary, strive to communicate with a human and willingly “go into the hands”.

In our experiment, 11 male rats from each line at the age of 2 months were studied. Biological material was collected and prepared, namely: 4 brain regions from 11 aggressive and 11 tame rats were dissected, and the total mRNA was isolated and purified as described in our works [21,22,23,24]. The obtained mRNA sequencing data was verified by semi-quantitative real-time PCR, as described in our works [21,22,23,24] and analyzed using bioinformatics methods as also described previously [21,22,23,24].

As a result of processing data from whole-genome sequencing, information was obtained on the levels of transcription of all active genes in the studied samples of 4 brain regions obtained from tame and aggressive rats. Table 2 shows the total number of reads and comparison with the reference genome for each brain region, and it also presents the total number of genes expressed in the 4 brain regions: at least 10 reads in each of them. Genes were considered as with differential between lines expression when the difference reached the 95% significance threshold. Transcriptomic profiles revealed significant differences between the aggressive and tame lines of rats for 44 genes in the hypothalamus, 42 genes in the hippocampus, 39 genes in the periaqueductal gray matter and 42 genes in the tegmental region of the midbrain (the total number of differentially expressed genes is 157, but after excluding the genes repeated in different brain regions, the total number of DEGs was reduced to 112).

### 4.2. Statistical Analysis

The total list of DEGs was subjected to principal component analysis (Varimax, maximization of variance): we performed this analysis using the PAST4.04 software package (Ref. [27] downloaded from the public website of the Natural History Museum pf the University of Oslo, Norway, namely: https://www.nhm.uio.no/english/research/resources/past/ (accessed on 15 January 2024)), in the bootstrap refinement mode via mode selection path “Multivariate”→”Ordination”→“Principal Components (PCA)”→“Correlation”→“Bootstrap” at 1000 rounds of the bootstrap verification with the option “Varimax” maximizing variance estimates.

### 4.3. Functional Analysis of DEGs and Construction of Gene Association Networks

Functional annotation of DEGs [identification of GO and KEGG terms (including metabolic pathways) enriched within the DEG list] was carried out with the help of Web service DAVID Bioinformatics Resources (Database for Annotation, Visualization and Integrated Discovery [46]). The DAVID software version 2021 (https://david.ncifcrf.gov/, accessed on 18 January 2024) was used with default settings: in search mode “Current Background: Rattus norvegicus” with respect to the reference rat genome RGSC Rnor_6.0 (UCSC Rn 6 July 2014).

Enrichment was examined both in relation to the results we obtained about transcriptomes of tame and aggressive rats and in relation to the default genome of gray rats in terms of KEGG PATHWAY and GO categories. Terms with a significance level of *p* < 0.05 were considered significantly enriched. We used the PANTHER database version 18.0 (https://pantherdb.org/, accessed on 18 January 2024) for a comparison of the gene ontology terms found.

The construction of gene networks based on interactions between the DEGs under study was performed by means of web service STRING-DB version 12.0 [47] with default settings (https://string-db.org/, accessed on 18 January 2024). We used pipeline of online computer tools for gene ontology analysis and gene network reconstruction for brain genes following recent trends in bioinformatics tool development.

## 5. Conclusions

In this work, we studied the transcriptomes of two unique lines of gray rats obtained after the long-term selection for behavioral reaction to humans. We identified and analyzed genes that change their expression in any of the four rat brain regions studied as a result of the artificial selection for tame or aggressive behavior.

Principal component analysis revealed only two significant factors contributing to gene expression variability in the studied brain regions. It was shown that the first component significantly correlates with the behavioral phenotypes, and the second significantly correlates with the specific genetic regulation of hippocampal function; DEGs were identified whose variability significantly correlates with each of the two factors.

The results of the functional enrichment analysis of DAVID and STRING complement each other and allow us to identify and describe key biological processes that participate in the formation of contrasting behavioral patterns of the two groups of gray rats. The genes engaged in broad network interactions have been identified. The results obtained confirm the presence of a multifactorial system for controlling the behaviors studied.

## Figures and Tables

**Table 1:** The distribution of all DEGs among GO and KEGG terms in the brain regions of tame and aggressive rats according to DAVID. Biological processes are presented that are significantly enriched within the total list of DEGs (tame rats compared to aggressive rats) (p_adj_ < 0.05 according to the binomial distribution test with Bonferroni’s correction for multiple comparisons).

Category	Term	Gene Symbols	N_GENE_	%	p_adj_
KEGG PATHWAY	Neuroactive ligand-receptor interaction	*Bdkrb2*,*Cckbr*,*Htr2c*,*Nmb*,*Pdyn*,*Prlr*,*P2rx4*,*Rln3*,*Sstr2*,*Tac3*,*Ucn*,*Vip*	12	10.9	0.005
UP KW PTM	Cleavage on pair of basic residues	*Alb*, *Cartpt*,*Enpp2*,*Pdyn*,*Rln3*,*Tac3*,*Ucn*,*Vip*	8	7.3	0.01
GO TERM BP DIRECT	Arachidonic acid secretion	*Bdkrb2*,*Nmb*,*Pla2g2c*,*Pla2g2d*,*Pla2g5*	5	4.5	0.01
UP KW BIOLOGICAL PROCESS	Lipid metabolism	*Aox1*,*Bdh1*,*Fuca1*,*Enpp2*,*Insig1*,*Liph*,*Mogat2*,*Pla2g2c*,*Pla2g2d*,*Pla2g5*, *Retsat*, *Thrsp*	12	10.9	0.01
GO TERM CC DIRECT	Extracellular region	*Alb*,*Defb17*,*Enpp2*,*Hspa1a*,*Hspa1b*,*Liph*,*Nmb*,*Pla2g2c*,*Pla2g2d*,*Pla2g5*,*Pdyn*,*Rln3*,*Tac3*,*Tecta*,*Ucn*,*Vip*	16	14.5	0.025
UP KW DOMAIN	Signal	*Alb*,*Cartpt*,*Cd22*,*Cspg4b*, *Defb17*,*Enpp2*,*Fat2*,*Fcgr3a*,*Fcgr2b*,*Fcrl2*,*Fjx1*, *Frem1*,*Fuca1*,*Htr2c*,*Htr3a*,*Lilrb3l*,*Liph*,*Lypd1*, *Mpeg1*,*Nmb*,*Pcdhga1*, *Pdia4*, *Pdyn*,*Pla2g2c*,*Pla2g2d*,*Pla2g5*,*Plod1*, *Prlr*,*Retsat*,*Rln3*,*Smoc2*,*Spint1*,*Sulf1*,*Tac3*,*Tecta*, *Ucn*,*Vip*, *Vstm2b*	38	34.5	0.025
UP KW PTM	Disulfide bond	*Alb*,*Bdkrb2*,*Cartpt*,*Cckbr*,*Defb17*,*Enpp2*,*Fat2*,*Fcgr3a*,*Fcgr2b*,*Fosb*,*Htr2c*,*Htr3a*,*Itm2a*,*Lilrb3l*,*Liph*,*Lypd1*,*Pla2g2c*,*Pla2g2d*,*Pla2g5*,*Pdia4*,*Pdyn*,*Prlr*,*P2rx4*,*Rln3*,*Sstr2*,*Tecta*, *Txnrd2*	27	24.5	0.05

Notes. N_GENE_: the number of DEGs; %: proportion of all DEGs; p_adj_: significance according to the binomial distribution test with Benjamini’s correction for multiple comparisons. Genes whose expression levels are significantly higher in tame rats are highlighted in blue, and genes upregulated in aggressive rats are red.

**Table 2:** Summary statistics for differentially expressed genes (DEGs) in the four brain regions: RNA-seq analysis was performed in three tame and three aggressive adult male rats (*Rattus norvegicus*).

Group	Tame vs. Aggressive Rats Brain Region			
	Hypothalamus	Hippocampus	PAG	MTg
Total number of sequence reads (NCBI SRA ID: PRJNA668014)	219,086,104	169,529,658	210,128,758	182,197,974
Reads mapped to reference rat genome RGSC Rnor_6.0, UCSC Rn6, July 2014 (%)	184,991,379 (84.4%)	146,521,467 (88.7%)	177,608,837 (84.5%)	158,310,590 (86.9%)
Expressed genes identified (at a threshold of at least 10 reads per gene in all 4 brain regions)	14039			
Statistically significant DEGs (p_ADJ_ < 0.05, Fisher’s Z-test with Benjamini correction)	44	42	39	42

## Data Availability

The primary RNA-Seq data analyzed in this work were deposited in the NCBI SRA database (ID = PRJNA668014) and processed in RatDEGdb (https://www.sysbio.ru/RatDEGdb/, accessed on 18 January 2024). Raw data are available upon request.

## Abbreviations

DEG	differentially expressed gene
ECR	extracellular region
GO	Gene Ontology
KEGG	Kyoto Encyclopedia of Genes and Genomes
MTg	tegmental region of the midbrain
PAG	periaqueductal gray matter
PCA	principal component analysis
PC1	first principal component
PC2	second principal component
TF	transcription factor
